# Factor XIII Deficiency With Repeated Severe Postoperative Bleeding After Laparotomy: A Case Report

**DOI:** 10.7759/cureus.60489

**Published:** 2024-05-17

**Authors:** Ryosuke Omoto, Yutaka Umemura, Atsushi Watanabe, Naoki Nakamoto, Satoshi Fujimi

**Affiliations:** 1 Emergency Department, Osaka General Medical Center, Osaka, JPN

**Keywords:** open abdominal management, postoperative bleeding, transmesosigmoid hernia, factor xiii concentrate, factor xiii

## Abstract

Patients with factor XIII (FXIII) deficiency present with a bleeding tendency that is difficult to diagnose because their coagulation test results are normal. We herein report a case of a 74-year-old male who presented to our hospital in cardiac arrest. After resuscitation, he was found to have sigmoid volvulus and necrosis; therefore, an emergency laparotomy was performed. Intraoperative findings revealed an extensive strangulated ileus in addition to sigmoid volvulus. We performed resection without reconstruction and maintained open abdominal management (OAM) for six days. After abdominal closure, the patient experienced postoperative bleeding four times from the mesenteric transection; three of the bleeding episodes required open hemostasis. Since he had mild coagulopathy during each bleeding episode, FXIII deficiency was suspected and diagnosed. After administration of FXIII concentrate, the tendency to intraoperative bleeding improved significantly. FXIII deficiency should be considered in cases of repeated severe bleeding, even when coagulation tests reveal no major abnormalities.

## Introduction

Factor XIII (FXIII) is a hemostatic protein that stabilizes blood clots and has a variety of functions in wound healing, tissue repair, hemostasis, and trauma [1]. Although FXIII deficiency has been reported to cause unexpected postoperative bleeding, it is rare and often difficult to diagnose because the prothrombin time (PT) and the activated partial thromboplastin time (aPTT) are within the normal range [1]. Therefore, postoperative bleeding is often treated as an unfortunate surgical complication, and a diagnosis of FXIII deficiency is rarely made [2]. In persistent perioperative bleeding associated with FXIII deficiency, there is growing evidence supporting the use of FXIII concentrates, despite varying guidelines [3-5]. In cardiac surgery, for example, reduced FXIII activity was reported to be independently associated with surgical re-exploration and increased 30-day mortality [6]. Administration of FXIII concentrates has been shown to be beneficial for patients with low FXIII levels, and early recognition and intervention may improve outcomes [1]. We herein report a case of FXIII deficiency that resulted in repeated severe postoperative bleeding after laparotomy.

## Case presentation

**Figure 1:**
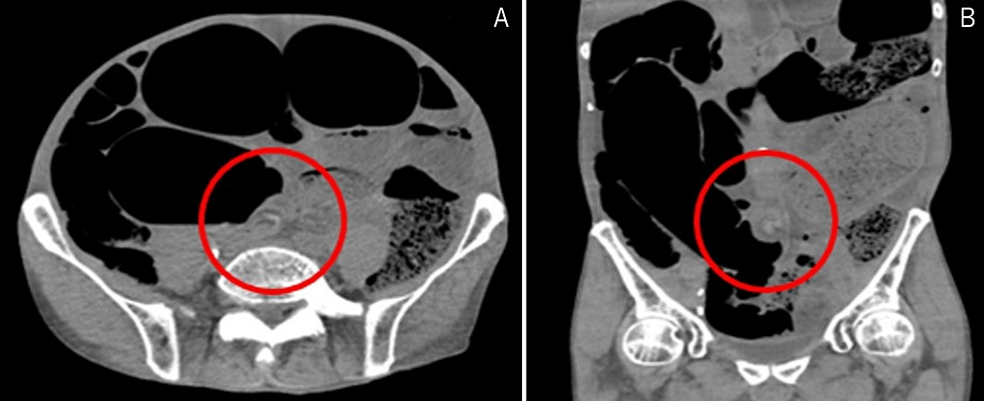
CT findings A and B showed a caliber change of the sigmoid colon, suggesting a sigmoid volvulus (red circle). CT: computed tomography

A 74-year-old male developed respiratory distress and was referred to the emergency medical service. His past medical history was significant for hypothyroidism without a previous bleeding history or surgical history. The patient experienced cardiopulmonary arrest shortly after contact. He was resuscitated five minutes after hospital arrival and was able to respond to commands. The duration of cardiac arrest was 20 minutes. His abdomen was distended, and computed tomography (CT) revealed a sigmoid volvulus (suspected to be the cause of cardiopulmonary arrest), which resulted in sepsis and respiratory failure due to diaphragmatic compression (Figure 1).

**Figure 2:**
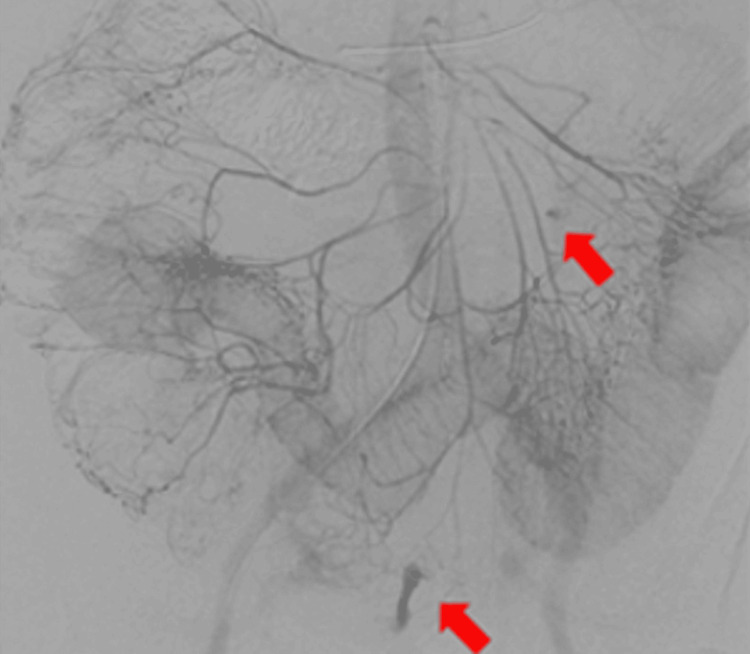
Angiography of the superior mesenteric artery Angiography of the superior mesenteric artery showed extravasations from the mesenteric edge of the small intestine (red arrow).

We attempted endoscopic detorsion of the sigmoid volvulus but abandoned it due to necrosis of the mucosal surface, and an emergency laparotomy was performed. Intraoperative findings revealed a sigmoid volvulus that had twisted 180° and become necrotic. The oral side of the colon was resected in descending order, and the anal side was resected in the rectosigmoid. We also found a transmesosigmoid hernia that resulted in extensive strangulation and necrosis of the small intestine, which was resected, leaving 100 cm from the Treitz ligament and 20 cm from the terminal ileum. Because the patient was hemodynamically unstable, we performed resection alone, without reconstruction, and maintained open abdominal management (OAM) using ABThera™, which separates the abdominal wall from the viscera, protects the abdominal contents, actively removes fluid, and reduces edema [7]. On day 3, an exploratory laparotomy was performed, and necrosis of the splenic flexure of the colon was found; therefore, we additionally resected it from mid-transverse to the end of the descending colon and continued OAM. On day 6, we successfully performed abdominal closure with end colostomy on the transverse side and end ileostomy of the ileum. After resuscitation, the patient continued to require high-dose catecholamines and continuous hemodialysis and filtration due to septic shock and persistent liver and kidney failure after cardiac arrest. On day 10, the patient's blood pressure suddenly decreased, and the abdominal drain became bloody. Contrast-enhanced CT showed extravasation from the mesenteric edge of the small intestine, and transcatheter arterial embolization was performed (Figure 2).

**Figure 3:**
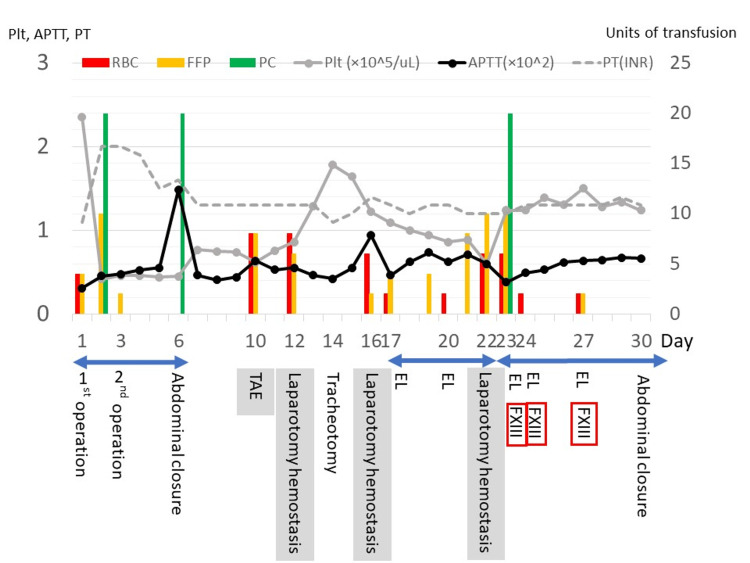
Trends in coagulation and platelet markers, transfusion volume, and surgical flow Trends in coagulation and platelet markers, transfusion volume, and surgical flow after admission are summarized along the timeline. Double arrowheads show the duration of open abdominal management. EL: exploratory laparotomy, Plt: platelet, aPTT: activated partial thromboplastin time, PT: prothrombin time, RBC: red blood cell, FFP: fresh frozen plasma, PC: platelet concentrate, INR: international normalized ratio, FXIII: factor XIII, TAE: transcatheter arterial embolization

On day 12, he experienced hemorrhagic shock again, and open hemostasis was performed. The operative findings showed that the mesentery of the descending colon, which had separated during the initial operation, was the cause of the bleeding. After hemostasis, abdominal closure was performed using tensile reduction sutures. On day 13, we performed a tracheotomy; however, the next day, diffuse postoperative bleeding occurred around the tracheostomy site, and we had difficulty stopping the bleeding. On day 16, he again experienced hemorrhagic shock, and laparotomy for hemostasis was performed. The cause of the bleeding was the mesenteric edge of the small intestine. After hemostasis, OAM was resumed. Since abnormalities in measurable coagulation markers, such as PT and aPTT, were mildly increased in each case of bleeding episodes and it had been some time since the initial operation, we considered that delayed wound healing was also related to the cause of the hemorrhage, and we examined the patient for suspected FXIII deficiency. On days 17 and 20, an exploratory laparotomy and OAM were performed. On day 22, the abdomen was successfully closed with stainless wire sutures; however, on the same day, oozing from the wire puncture sites persisted and was uncontrolled, and we performed open hemostasis and resumed OAM. The FXIII activity measured on day 16 was found to be 16% on day 22, while the von Willebrand factor and antiphospholipid antibody levels were normal. On days 23, 24, and 27, an exploratory laparotomy was performed with 720 units of FXIII concentrate (Fibrogammin PⓇ), and the intraoperative bleeding markedly improved. The activity of FXIII before the administration of FXIII concentrate on day 24 was 47%. After administration of the FXIII concentrate, the abdomen was successfully closed on day 30 without hemorrhagic events. However, the patient died of multiple organ failure due to sepsis and cardiac arrest on day 38. Trends in coagulation and platelet markers, transfusion volume, and surgical flow after admission are summarized in a time course chart (Figure 3).

During the first month after resuscitation, blood pressure support required norepinephrine, and the patient presented shock vitals during postoperative bleeding, but, otherwise, his blood pressure was stable. His renal function was also persistently anuric, so continuous hemodialysis and filtration (CHDF) was introduced on the second day of admission, and the patient was unable to get off CHDF until his death.

## Discussion

FXIII is a coagulation factor that stabilizes fibrin; when tissue injury occurs and fibrin clots form, FXIII is rapidly activated by thrombin to become FXIIIa, which cross-links fibrin and stabilizes the clot [1]. FXIII also plays an important role in wound healing and tissue repair and exerts antifibrinolytic activity through cross-linking of α2-antiplasmin to fibrin [1].

As FXIII is an enzyme outside the framework of the coagulation cascade, FXIII deficiency does not cause abnormalities in PT and aPTT [1]. Therefore, postoperative bleeding is often treated as an unfortunate event, and a diagnosis of FXIII deficiency is rarely made [2]. However, when wound healing is unusually delayed or postoperative bleeding recurs unexpectedly in the absence of major abnormalities in coagulation capacity, FXIII deficiency should be suspected [1]. In our case, FXIII deficiency was suspected on day 16, but earlier intervention may have been possible if it had been suspected earlier.

The normal level of FXIII activity is 70%-130%; FXIII deficiency is diagnosed when it is less than 70% [1]. FXIII deficiency is classified into congenital and acquired causes, with acquired FXIII deficiency being classified as either inhibitor-induced or secondarily decreased (caused by hypo-synthesis due to liver dysfunction or hyper-consumption following trauma or surgery) [8]. Congenital FXIII deficiency is an autosomal recessive inherited disease with a prevalence of about one in 1-3 million. The prevalence varies by region, with higher rates in regions with high rates of consanguineous marriages, but there are no global differences in the ethnic or racial groups affected [9]. Acquired FXIII deficiency, on the other hand, is very rare, and its prevalence is unknown. In our case, since blood tests for inhibitors or autoantibodies of FXIII were not available at our hospital, it is not certain whether immunological mechanisms were at work. Hyper-consumption due to repeated surgical invasions and hypo-synthesis due to liver dysfunction resulting from short bowel syndrome might have contributed to the decrease in FXIII. However, since PT and aPTT were within the range of mild coagulopathy due to a large amount of FFP transfusions and the activity of FXIII was markedly decreased to 16%, we speculate that an immunological mechanism was most likely at work in the reduction of the FXIII factor.

In our case, although we performed precautionary measures in all surgeries, such as preoperative FFP or platelet administration and careful intraoperative hemostasis using ultrasonic coagulation cutting devices, sutures, and ligations, we had difficulty with postoperative hemostasis, which required multiple reoperations. After approximately three weeks of hospitalization, the patient was diagnosed with FXIII deficiency and administered FXIII concentrates, and bleeding was controlled, but the patient's prognosis was unfavorable. An early diagnosis and intervention of FXIII deficiency may have changed the course of the patient. Our case highlights the importance of considering undiagnosed FXIII deficiency.

Postoperative bleeding events and blood transfusions are significantly more frequent in patients with FXIII deficiency [3-5]. In a previous observational study, a multivariate analysis revealed that reduced FXIII activity was independently associated with surgical re-exploration and increased 30-day mortality [6]. In neurosurgery, FXIII levels of <60% were an independent risk factor for postoperative bleeding [3,4].

As mentioned above, the association between perioperative bleeding and FXIII has been reported in many studies, but recommendations for the use of FXIII concentrate in clinical guidelines are still controversial and limited [10,11]. The European Society of Anaesthesiology recommends the administration of FXIII concentrate (30 IU/kg) in the clinical setting of ongoing or diffuse bleeding and low clot strength despite adequate fibrinogen concentrations if FXIII activity is critically reduced (i.e., <60% activity) [10]. On the contrary, the International Society of Thrombosis and Hemostasis recommends against the use of FXIII concentrates in the management of perioperative bleeding because the benefit in acquired FXIII deficiency has not yet been proven [11].

The limited recommendation for FXIII therapy in clinical guidelines suggests a lack of awareness of the potential for FXIII deficiency in postoperative bleeding and delayed wound healing. However, the most recent review indicates that the administration of FXIII concentrates is beneficial for patients with a low FXIII level [1]. In cases of repeated or uncontrolled bleeding, increased awareness of FXIII testing can help identify patients with acquired FXIII deficiency. Further clinical studies are essential to clarify the efficacy of FXIII therapy; however, in the setting of persistent perioperative bleeding, the administration of FXIII concentrates should probably be considered only when decreased FXIII activity is detected.

## Conclusions

We encountered a case of FXIII deficiency with repeated severe postoperative bleeding after laparotomy. FXIII deficiency should be considered in cases of repeated postoperative bleeding in patients with normal coagulation or mild coagulopathy. Early recognition and intervention can potentially improve outcomes in patients with FXIII deficiency-related bleeding events. Administration of FXIII concentrates may be beneficial for controlling perioperative and postoperative bleeding in patients whose FXIII activity is significantly low. Further studies and increased awareness among clinicians are needed to better understand and manage FXIII deficiency in surgical settings.
